# Enhancing causal inference in population-based neuroimaging data in children and adolescents

**DOI:** 10.1016/j.dcn.2024.101465

**Published:** 2024-10-19

**Authors:** Rachel Visontay, Lindsay M. Squeglia, Matthew Sunderland, Emma K. Devine, Hollie Byrne, Louise Mewton

**Affiliations:** a The Matilda Centre for Research in Mental Health and Substance Use, The University of Sydney, Sydney, Australia; b Department of Psychiatry and Behavioral Sciences, Medical University of South Carolina, USA

**Keywords:** Causality, Causal inference, Propensity scores, Neuroimaging, Children, Adolescents

## Abstract

Recent years have seen the increasing availability of large, population-based, longitudinal neuroimaging datasets, providing unprecedented capacity to examine brain-behavior relationships in the neurodevelopmental context. However, the ability of these datasets to deliver *causal* insights into brain-behavior relationships relies on the application of purpose-built analysis methods to counter the biases that otherwise preclude causal inference from observational data. Here we introduce these approaches (i.e., propensity score-based methods, the ‘G-methods’, targeted maximum likelihood estimation, and causal mediation analysis) and conduct a review to determine the extent to which they have been applied thus far in the field of developmental cognitive neuroscience. We identify just eight relevant studies, most of which employ propensity score-based methods. Many approaches are entirely absent from the literature, particularly those that promote causal inference in settings with complex, multi-wave data and repeated neuroimaging assessments. Causality is central to an etiological understanding of the relationship between the brain and behavior, as well as for identifying targets for prevention and intervention. Careful application of methods for causal inference may help the field of developmental cognitive neuroscience approach these goals.

## 1 Introduction

Establishing causality is central to an etiological understanding of the relationship between the brain and behavior, as well as for identifying targets for the prevention and intervention of adverse outcomes in childhood and adolescence. However, many neuroimaging studies to date have been limited by cross-sectional designs, small sample sizes, and analytic methods that produce only *correlational* information. Study designs and analytic methods capable of yielding causal insights have not been routinely applied in the field. Arguably, this ‘causality gap’ (Siddiqi et al., 2022) has held back the successful conversion of pediatric neuroimaging findings into robust therapeutic targets.

Promisingly, recent years have seen the increasing availability of large-scale, population-based, longitudinal neuroimaging datasets (i.e., ‘population neuroscience’). The observational data derived from these studies are ideally suited to longitudinal analyses of neurobiological predictors and consequences of behavior and have been used extensively to explore developmental phenomena (Kievit et al., 2022, King et al., 2018). Increasingly, data analytic methods derived from other fields (e.g., developmental psychology; King et al., 2018) have been applied to these data to yield novel insights into brain-behaviour relationships across development (Brown et al., 2015, Schumann et al., 2010, Volkow et al., 2018). With successful dissemination and application of appropriate analytical methods established in other fields like econometrics, these longitudinal datasets represent an opportunity to examine *causal* brain–behavior relationships across development.

These methods to enhance causal inference require sufficient sample size and robust measurement over multiple waves of data collection, and are increasingly being applied to epidemiological survey data more broadly (Watkins, 2019). With the establishment of several recent child and adolescent neuroimaging cohorts, such as the Adolescent Brain Cognitive Development (ABCD), National Consortium on Alcohol and NeuroDevelopment in Adolescence (NCANDA), and IMAGEN studies (Brown et al., 2015, Schumann et al., 2010, Volkow et al., 2018), these methods can now be applied to better understand causal brain–behavior relationships during this critical developmental period.

We note that the ABCD, NCANDA, and IMAGEN cohorts were all (at least partially) established with the aim of studying the relationship between substance use and brain development. This focus reflected growing interest in understanding how substance use, which is typically initiated during adolescence, is related to neurodevelopment (Lees, Garcia, et al., 2021) and how this information can be used to inform substance use prevention and intervention (Cservenka et al., 2015, Rezapour et al., 2024, Volkow and Boyle, 2018). With this in mind, the current paper uses examples which focus on substance use, and particularly alcohol use, which is the most commonly used substance by youth.

### 1.1 Obstacles to causal inference

For a relationship to be a causal one, manipulating (or intervening on) an exposure (or risk factor) must lead to changes in an outcome. Identifying that a brain structure or network, for example, has a causal effect on a behavior provides evidence that an intervention focused on this brain structure or network would lead to changes in the relevant behavior. In the reverse, if a behavior has a causal effect on a brain structure or network, we then have evidence that intervening on this behavior would lead to changes in that brain structure or network.

Several sources of bias present challenges to making causal inferences when using observational neuroimaging data, i.e., our confidence that an observed relationship reflects a genuine causal effect of an exposure on an outcome. While measurement error and selection bias pose their own threats to causal inference, the primary challenges that researchers grapple with are determining whether an observed association spuriously arises from the influence of other variables related to both the exposure and the outcome – i.e., ‘confounding’ (Ohlsson and Kendler, 2019), and further, isolating the effect in the direction of interest.

**Fig. 1:**
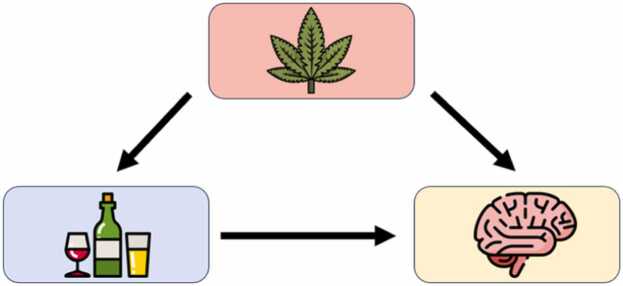
An example of a confounded relationship in pediatric neuroimaging: the effect of alcohol use on brain development is confounded by cannabis/other drug use (images: Flaticon.com).

For example, cannabis/other drug use is related to both alcohol use and certain MRI-ascertained adolescent brain measures (e.g., structural differences in frontal and parietal regions; Chye et al., 2020; Lees, Debenham, et al., 2021). This means that when investigating the effect of alcohol use on brain development in adolescence, this relationship may be confounded by cannabis/other drug use. This is represented in Fig. 1 in a basic *directed acyclic graph* (DAG) – a graphical depiction of assumed causal relationships in which all links between variables are arrows reflecting causation in one and only one direction (i.e., directed), and in which there are no feedback loops (i.e., acyclic; Kunicki et al., 2023).

There are also challenges here related to directionality – that is, ascertaining whether the observed measures are the cause or consequence of a given behaviour and/or symptom. For example, while many studies have identified a link between alcohol use and grey and/or white matter volumes in adolescents (Squeglia et al., 2014), it is difficult to determine whether these neurophysiological changes are driven by alcohol use, or whether such features are predictive markers of alcohol use in this population. In our example it is likely that there is reverse causation from brain structure/function to alcohol use in addition to a causal effect in the direction of interest, making it difficult to isolate the latter (this could be depicted in a DAG in a longitudinal context; see Kunicki et al., 2023). The problem of directionality extends to the field more generally, with most neuroimaging methods only measuring neurophysiological correlates of behaviour and/or symptoms. These challenges are particularly relevant to cross-sectional imaging studies, where the dynamics of such relationships are unable to be explored over time.

### 1.2 Causal inference and the experimental method

The dominant framework in the causal inference method is based on the concept of ‘potential outcomes’ or ‘counterfactuals’ (Rubin, 1974). This framework, also known as the ‘Rubin causal model’ is based on the idea of counterfactual outcomes under alternative exposures to conceive of causal effects at the level of the individual – in other words, what *would* have happened under an alternative exposure. Accordingly, a definition of a causal effect in this framework is that an exposure has a causal effect for an individual if the outcome that occurred for that individual under one exposure does not equal the outcome that would have occurred under another.

It follows that the ideal method for establishing causality would involve two identical worlds that differ only in one respect: in one world, everyone is exposed to a given variable, whilst in the other, no-one is exposed to this same variable. If our outcome of interest were to differ between these two worlds, then we could definitively claim that our exposure causes our outcome. While this ideal method is impossible to realise, the experimental method (including randomized experiments and randomized controlled trials) is its closest approximation. Randomization attempts to eliminate confounding through the creation of groups that can be considered comparable at baseline, with the only difference between the groups being the introduction of an intervention or exposure variable. Randomizing an exposure should, in theory, ensure the exposure is not associated with covariates, nor associated with past levels of the outcome of interest, avoiding the problems of confounding and reverse causation. For this reason, the experimental method is considered the gold-standard for assessing causality.

However, as previously noted (Hamaker et al., 2020), experiments are often not feasible or ethical in developmental cognitive neuroscience. Take again, for example, research questions involving the effects of alcohol use on brain structure or function. Conducting randomized controlled trials which manipulate long-term alcohol exposure has proven near impossible in adults for a variety of reasons, including ethical concerns (ethanol is a known carcinogen and increases all-cause mortality; Kassaw et al., 2024; Millwood et al., 2023; Zhao et al., 2023) and logistical obstacles (compliance with long-term assigned drinking conditions). Further, manipulating alcohol exposure in children and adolescents would be ethically indefensible.

Similarly, many relationships in the reverse direction, for example, how differences in brain structure or function impact subsequent substance use behaviors, are less amenable to a randomized controlled trial framework. Brain stimulation techniques such as transcranial magnetic stimulation (TMS) and transcranial direct current stimulation (tDCS), which use magnetic currents to modulate cortical activity, are some of the primary non-invasive methods for manipulating brain function to examine effects on subsequent behavior (Mostafavi et al., 2020). These methods are being increasingly applied to the alcohol use context, with some showing therapeutic promise in reducing cravings and consumption in those with alcohol use disorder (e.g., Belgers et al., 2022). However, due to limited sample sizes and variances in methodology (Mostafavi et al., 2020), it is difficult to draw strong conclusions concerning the utility of these methods in a treatment capacity (Petit et al., 2022).

Further, such methods could not ethically be applied to explore the inverse relationship – that is, whether stimulation of certain brain areas leads to *increased* alcohol consumption or alcohol misuse behaviours (e.g., to corroborate existing observational evidence indicating that lower gray matter volume in brain regions linked with reward processing and executive function is associated with substance use initiation in adolescence; Lees, Garcia, et al., 2021; Squeglia and Cservenka, 2017).

Because of these limitations, researchers in the field of developmental cognitive neuroscience largely rely on observational data to understand brain-behavior relationships. However, analytic innovations in causal inference mean that this need not be a total barrier to developing causal knowledge.

## 2 The Target Trial framework

While it was long-regarded impossible to use observational data for causal inference (Hernán, 2018), a recent push for ‘target trial’ emulation using observational data is beginning to change epidemiological orthodoxy. Within the target trial framework, a researcher must conceive of an ideal trial (in a hypothetical world in which practical and ethical obstacles do not exist) that would be conducted to answer a given research question. Then, the researcher conducts analysis of observational data in a way that corresponds as closely as possible to that target trial (Hernán and Robins, 2020). Key here is the specification of all the factors relevant to a randomized trial protocol, such as eligibility criteria, exposure definition, interval between exposure and follow-up, and comparisons of interest (Hernán and Robins, 2016). These components of the target can then be explicitly emulated using observational data, mimicking many of the desirable properties of randomized controlled trials (see Table 1 for an example).

**Table 1:** Specification of components in a target trial emulation for investigating the effects of alcohol use on adolescent brain outcomes.

**Component**	**Hypothetical target trial**	**Emulation using observational data**
Aim	To estimate the effects of persistent binge drinking (5+ drinks/occasion) on adolescent brain health	Same
Eligibility	Adolescents aged 12–13	Adolescents aged 12–13, with exclusion of individuals reporting past 6-month sub-binge drinking at any measurement wave
Treatment strategy	Participants are randomly assigned to either: 1) Regular binge drinking over 2 years 2) No alcohol	Repeatedly categorized into 1) or 2) at yearly measurements (x3), based on past 6-month alcohol consumption
Assignment procedures	Randomization to one of the 2 treatment strategies	To emulate random assignment, confounders are adjusted for via G-methods or targeted maximum likelihood estimation*
Outcome	Cortical gray matter volume	Same
Follow-up period	Starts at randomization and finishes 2.5 years later at time of MRI scan	Starts at study baseline (when individuals first report on past 6-month alcohol consumption) and finishes 2.5 years later at time of MRI scan
Causal contrasts of interest	Per-protocol effect: effect had all individuals adhered to their assigned drinking level	Observational analogue of the per-protocol effect (i.e., using observed data of time-varying drinking patterns to generate predictions about *hypothetical* stable drinking trajectories** that correspond to the trial conditions)
Analysis plan	Compare mean gray matter volume between randomized groups, excluding those who do not adhere to assigned drinking level and using inverse probability of censoring weights to account for differential attrition*	Compare mean predicted gray matter volume corresponding to persistent binge drinking and persistent abstinence from alcohol and using inverse probability of censoring weights to account for differential attrition, if applicable*

**Note that target trial emulation is also possible for single point in time (baseline) exposures, but that in the context of neurodevelopment it is often the cumulative effects of an exposure that are of interest. It is also possible to use target trial emulation for dynamic exposures regimens.

* See Section 3 for these methods

As is evident in Table 1, a key element of target trial emulation is approximating the random assignment mechanism to deal with confounding. Traditional statistical methods for confounder control (e.g., regression with adjustment for covariates) could *theoretically* address confounding bias in the target trial framework in very simple cases. For example, when there are a small number of plausible covariates, the researcher has confidence in the functional form of their relationship with the outcome, and it suffices to measure the exposure, covariates, and outcome just once. However, this set of circumstances is rare, motivating the development of alternative methods that have been purpose built to enhance valid causal inference (Chan et al., 2024). Here we aim to introduce key methods for improving causal inference from observational data, and review how and to what extent these methods have been applied to analyses of large-scale, population-based neuroimaging data in children and adolescents to date.

## 3 Key approaches to improve causal inference

Hammerton and Munafo distinguish between two general families of methods for enhancing causal inference: alternative “design-based” approaches (also called ‘natural experiments’ or ‘quasi-experiments’) and statistical ones (Hammerton and Munafò, 2021). Design-based analyses attempt to mitigate bias *by design*, i.e., they select particular variables (e.g., instrumental variables which proxy for the exposure of interest) or cohorts (e.g., related individuals for use in within-family analyses) that are inherently less susceptible to confounding and/or other biases. While powerful methods, these additional data features are often difficult to achieve (e.g., proxies may not make sense for certain exposures, and related individuals can be hard to recruit).

By contrast, statistical approaches apply enhanced analytical techniques to mitigate bias while still using conventional cohort or case-control data. Several statistical methods have been developed for enhancing causal inference from observational data that aim to better mimic the randomization mechanism of randomized experiments. The key methods here are those based on propensity scores, generalized methods or ‘G-methods’ (including marginal structural models, G-computation, and G-estimation of structural nested models), and targeted maximum likelihood estimation, which are discussed in detail below.

Importantly, two core assumptions must be met to make valid causal inferences from any of these statistical methods: exchangeability and stable unit treatment value. Conditional exchangeability means that risk of an outcome in the binge drinking group would have been the same for the abstaining group, had those in the binge drinking group abstained from alcohol (Hernán and Robins, 2020). That is, *conditional* on the measured confounders, individuals in each group should be exchangeable. Practically, this means there can be no unmeasured confounding; valid causal inference is therefore reliant on the inclusion and accurate measurement of *all* relevant confounders – in practice, a difficult assumption to fulfill and verify. Stable unit treatment value requires two things: that there cannot be multiple versions of a given exposure group (which may not hold in the case of binge drinking due to different volume of consumption in a session or frequency of drinking sessions), and that outcomes do not vary with changes to the exposure received by other individuals – i.e., there is no interference amongst individuals; Rubin, (1980)).

### 3.1 Propensity score-based methods

A propensity score captures the probability of a given exposure for an individual given their values on all relevant covariates, thus reducing often highly dimensional covariate data into a single score (Benedetto et al., 2018, Brookhart et al., 2013). Propensity scores can be calculated simply, often involving a logistic regression that models the exposure of interest as the outcome with all relevant covariates as predictors. Once the propensity score has been generated, it can be used for matching, stratification, as a covariate for adjustment in regression, or to weight a regression. This last approach is often known as inverse probability of treatment weighting, in which individuals are weighted by the inverse of their propensity to have the exposure they did given their specific covariate profile. Of note for longitudinal cohort studies involving children and adolescents, where non-random attrition over study waves is common, inverse probability of censoring weights can also be calculated to account for the resultant selection bias. Inverse probability of treatment and censoring weights can then be combined into a single weight that adjusts for both confounding and selection bias.

**Fig. 2:**
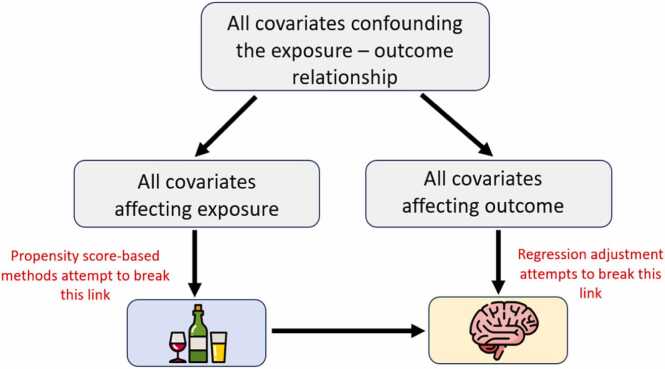
A depiction of how methods based on propensity scores and regression adjustment control for confounding variables. Note that if either the effects of covariates on exposure or the effects of covariates on outcome are removed, confounding is successfully controlled for.

Methods based on propensity scores address confounding bias by breaking the association between relevant covariates and the exposure of interest. This contrasts with traditional regression adjustment, which aims to break the association between relevant covariates and the outcome of interest (see Fig. 2). A key benefit of propensity score-based methods is dimensionality reduction (meaning they can reduce large numbers of covariates into a single score; Greenland et al., 2016, Melberg, 2006, Thoemmes and Ong, 2016), simplifying analysis and interpretation. Another advantage over regression adjustment is that these methods are not reliant on correctly modelling covariate-outcome relationships (i.e., the algebraic form specified in the regression model). Instead, the propensity score model must correctly specify the form of the relationships between covariates and exposure. However, this is an easier task given it is possible to include higher order terms and interactions without regard for collinearity or overfitting (this is because the propensity score model is not built for interpretation, and need not be generalizable; Beal and Kupzyk, 2014).

One limitation is that balance on propensity scores does not necessarily guarantee balance on the underlying covariates, and balance may be particularly hard to achieve in longitudinal extensions of propensity scores (see Section 3.2). However, in contrast to standard regression adjustment, propensity score models can be checked for how well they achieve the desired balance of covariates across the different levels of exposure, and respecified to attempt to improve fit. There are now also estimators specifically designed to maximise covariate balance (e.g., the ‘covariate-balanced propensity score’; Imai and Ratkovic, 2014). While it is still possible to misspecify the relationship between covariates and exposure probability, propensity score-based methods can be combined with traditional regression adjustment to increase the chances of valid causal inference – only one of either the covariate–exposure or covariate–outcome relationships need be correctly specified to control for confounding (a property known as ‘double-robustness’).

A further criterion for valid causal inference using propensity scores – and all causal inference methods that work by modelling the exposure – is the positivity assumption: that each individual has a non-zero probability of being both exposed and unexposed. In our example, positivity would require that for any given combination of covariate values there are individuals belonging to both the bingeing and non-bingeing categories (as is the case in randomized trials, where it is possible for any individual to be assigned to any condition for any combination of covariate values). This means that high-dimensional data poses problems for conventional regression adjustment *and* propensity score-based methods alike: for the former there is likely extreme extrapolation when regressing outcome on predictors, while for the latter there is likely extreme extrapolation when generating propensity scores, which in turn will bias any effect estimates (Imbens, 2004).

### 3.2 The ‘G-methods’

Methods based on propensity scores can effectively deal with confounding bias and improve causal inference in analyses exploring temporally distinct exposure, covariates, and outcome which have each been measured once (i.e., exposure and covariates measured at baseline, and outcome measured at a later time-point). However, as captured in the target trial example presented in Table 1, in the context of neurodevelopment it is often the cumulative effects of an exposure over a prolonged period that are of interest. Using observational data, researchers must contend with time-varying exposures as well as time-varying covariates that are affected by past exposure, such that they act as confounders and mediators over time. For example, cannabis use at some time during the exposure period may be a common cause of later alcohol consumption and brain outcomes, and may itself be affected by past alcohol consumption.

The so-called generalized methods or ‘G-methods’ were created to deal with more dynamic confounding bias that may vary over time, in addition to any stable confounders. Regression adjustment sets or ‘fixes’ the values of all confounders, including time-varying ones. This means that where part of the effect of an earlier exposure is mediated by one or more of these confounders (i.e., a mediated effect; see Box 1 for a graphical depiction), this part of the effect will be removed from the estimate. In contrast, in different ways, the G-methods do not fix values of these variables, allowing for mediated effects while still controlling for confounding (Hernán and Robins, 2020, Mansournia et al., 2017, Naimi et al., 2017). The parameters derived from these models can be used to estimate and compare the effects of any stable or dynamic trajectories of an exposure (e.g., drinking status). The three G-methods are marginal structural models, G-computation, and G-estimation of structural nested models.

Box 1 **Marginal structural model example.**

**Table:** 

A DAG depicting example relationships between variables over time. Note that cannabis leaves represent all time-varying covariates at a given measurement occasion, and that baseline time-fixed covariates at are also modelled but are not shown in this illustration for clarity.	
In this example, a researcher wants to compare the effects of consistent binge drinking vs consistent abstinence across all waves. The main outcome is cortical gray matter volume at time 6 (t6). To account for confounding, a marginal structural model would weight each individual by the product of three inverse probability of treatment weights. The first inverse probability of treatment weight reflects inverse probability of drinking status at t1 given time-fixed covariates (e.g., sex and parental education status). The second weight reflects inverse probability of drinking status at t3 given time-fixed covariates, drinking status at t1, and time-varying covariates at t2 (e.g., other drug use, psychopathology, and MRI data).	Finally, the third weight reflects inverse probability of drinking status at t5 given time-fixed covariates, drinking status at t1 and t3, and time-varying covariates at t2 and t4. A product of these weights is then used to weight a regression of the outcome (cortical gray matter volume) on t1, t3, and t5 drinking status. The estimated parameters can finally be used to produce causal estimates corresponding to hypothetical drinking status trajectories – in this case, by predicting the difference in outcome when t1, t3, and t5 are set to abstinence and when t1, t3, and t5 drinking are set to binge drinking.

The most frequently implemented of the G-methods are marginal structural models – an extension of inverse probability of treatment weights. A marginal structural model analysis is weighted by a single weight that is the product of inverse probability of treatment weights generated sequentially at each wave of exposure (this final weight can also be combined with sequential inverse probability of censoring weights to address attrition bias). This is perhaps the simplest statistical method for accounting for confounding that, unlike conventional regression adjustment, still allows for mediated effects in a longitudinal context. For a worked example and figure refer to Box 1.

Alternatively, using all observed data, G-computation first regresses the outcome on exposure and covariates, and then uses this model to simulate individual outcomes in two hypothetical worlds (in the case of a simple, binary exposure). This mimics the ideal experiment introduced previously: in one world, each individual is evaluated as if exposed, and in the other, each individual is not exposed. The difference between these hypothetical outcomes (averaged across individuals) is considered the causal estimate (Chatton and Rohrer, 2023, Robins, 1986). In a complex longitudinal context, G-computation accounts for dynamic variables by incorporating the distribution of covariates over time, modelling sequentially from earliest wave to last. Note that in most situations with complex longitudinal data, predicting time-varying covariates and outcomes in the chosen exposure scenarios will require Monte Carlo simulations.

As G-computation is based on a regression of outcome on exposure and covariates, it can be combined with a propensity score-based method to achieve double-robustness, for example with inverse probability weights via what is known as ‘doubly-robust standardization’ or ‘augmented inverse probability weighting’ (Chatton and Rohrer, 2023, Clare, 2020, Vansteelandt and Keiding, 2011). The simulation mechanism of G-computation can also be extended to simulate outcomes for censored individuals, accounting for attrition. However, it should be noted that G-computation is vulnerable to the ‘g-null’ paradox, i.e., the null may be rejected with large samples even when it is true. For a worked example and figure refer to Box 2.

Box 2 **G-computation example.**

**Table:** 

In this example, a researcher again wants to compare the effects of consistent binge drinking with consistent abstinence across all waves, using the same data depicted in Box 1. First, the observed data are used to model cortical gray matter volume given binge drinking status at t1, t3, and t5 and time-varying covariates at t2 and t4 (note that time-fixed covariates are also modelled but are not shown in this illustration for clarity). The observed data are also used to model each time-varying covariate at both t2 (given t1 exposure and time-fixed covariates) and t4 (given t1, t3 exposure and time-fixed, t2 covariates). Then, the parameters estimated from these models are used to simulate the time-varying covariates over the course of the study for each individual under two different scenarios – one in which all individuals drink at each time-point, the other in which all individuals abstain at each time point. Subsequently, brain outcomes are estimated under these two scenarios. Specifically, time-varying covariates at t2 are simulated for each individual corresponding to scenarios of t1 binge drinking and no t1 binge drinking. Then, time-varying covariates at t4 are simulated corresponding to scenarios of t1 and t3 binge drinking and no t1 or t3 binge drinking, based also on the corresponding simulated t2 time-varying covariates. Finally, cortical gray matter volume at t6 is simulated for the scenarios corresponding to t1, t3, and t5 binge drinking and no t1, t3, or t5 binge drinking, based also on the corresponding simulated t2 and t4 time-varying covariates.
The difference between the mean predicted outcome across the two scenarios is the causal estimate.

Finally, there is G-estimation of structural nested models, where multi-wave data are treated like nested ‘trials’. As opposed to G-computation, which works forward from the first wave to the last, G-estimation works backward from the last wave at which exposure was measured, sequentially modelling the outcome while adjusting for covariates and exposure values from previous waves (Picciotto and Neophytou, 2016). Working backward like this ensures that the effects of later exposure/covariates are removed when modelling the effects of an exposure at any given time, avoiding the need to fix time-varying covariates (Vansteelandt and Joffe, 2014). Unlike marginal structural models and G-computation, the sample must first be weighted by inverse probability of censoring weights and limited to uncensored individuals to account for selection bias from differential attrition *prior* to performing G-estimation. For a worked example and figure refer to Box 3. Doubly-robust extensions for G-estimation have also been proposed (Daniel et al., 2013).

Box 3 **G-estimation of structural nested models example.**

**Table:** 

In this example, a researcher again wants to compare the effects of consistent binge drinking with consistent abstinence across all waves, using the same data depicted in Box 1. Causal effects of binge drinking at any given time on brain outcomes can be isolated by sequentially modelling the relationship backward from the last wave to the first. In a) the researcher models the effect of binge drinking at t5 on cortical gray matter volume at t6, adjusting for binge drinking at t1 and t3, and time-varying covariates at t2 and t4. Using the generated parameters, the estimated effect of binge drinking at t5 is then removed from the t6 outcome for each individual who did binge drink at t5, creating a new outcome variable that simulates what would have happened if all individuals abstained from binge drinking at t5. Then in b), the researcher models the effect of binge drinking at t3 on this new, simulated outcome variable, adjusting for binge drinking at t1 and time-varying covariates at t4. Again, the generated parameters can be used to remove the effect of binge drinking at t3 from the outcome simulated in a), creating a new outcome variable that simulates what would have happened if all individuals abstained from binge drinking at t3 and t5. Finally, in c), the researcher can model the effect of binge drinking at t1 on the simulated outcomes from b). At the end of this process, the researcher has separate effect estimates for binge drinking at each of t1, t3, and t5, which can be combined to compare the effects of consistent binge drinking with consistent abstinence. A visual representation of structural nested models, adapted from Picciotto and Neophytou (2016). Note that time-fixed covariates are also modelled but are not shown in this illustration for clarity. Dashed boxes reflect the exposure–outcome pair being estimated at a given time.

### 3.3 Targeted maximum likelihood estimation

Targeted maximum likelihood estimation is another kind of doubly-robust estimator, with the advantage that, unlike other doubly-robust methods (Schuler and Rose, 2017), it optimizes bias-variance trade-off (bias referring to underfitting the model and failing to accurately capture the relationship; variance referring to overfitting the model and potentially identifying relationships that do not exist). It is easier to implement than augmented inverse probability weighting and generally demonstrates unbiased results (Clare, 2020).

Targeted maximum likelihood estimation requires estimation of the same two models described thus far – an exposure model based on covariate values (propensity score model), and a regression of outcome on covariate values. However, these models are used differently. To compare the effects of drinking and abstinence on a brain outcome, the generated outcome model is used to predict the outcome for each individual assuming that all individuals drink, and then separately assuming that all individuals abstain (comparable to G-computation). The propensity score model is then employed to generate a ‘clever covariate’ – an inverse probability weight for those that drank, and a negative inverse probability weight for those that did not. Then a new model is generated in which the outcome is regressed solely on the clever covariate with the initially predicted outcome supplied as a fixed intercept. The coefficient estimated by this regression is subsequently used as a fluctuation parameter – i.e., a parameter to be added to the initial outcome models to update or ‘tune’ the expected outcome estimates under drinking and abstinence (Van der Laan and Rose, 2011). The causal estimate is then generated by taking the difference between these two estimates. Again, inverse probability of censoring weights can be used in the initial outcome model to account for differential attrition. Targeted maximum likelihood estimation can be extended to cases with dynamic exposure and covariate values using sequential estimation that, like with G-estimation, starts from the final time point and iterates backward.

### 3.4 Causal mediation analysis

Causal mediation analysis also warrants discussion in the developmental cognitive neuroscience context. It is not strictly interested in the exposure–outcome relationship (and therefore not explicitly focused on mimicking the randomization mechanism), but rather, the extent to which that relationship is *mediated* by another variable of interest. Causal mediation extends traditional mediation analysis methods, allowing for the decomposition of total effect into direct and indirect effect even in the presence of exposure–mediator interaction and non-linearity (Pearce and Lawlor, 2017, Richiardi et al., 2013, VanderWeele, 2016). It also involves explicitly stating and testing underlying assumptions, e.g., that there is no unmeasured confounding. Causal mediation analysis can be combined with G-computation to account for time-varying exposures, mediators, and confounders (‘mediational G-formula’; VanderWeele and Tchetgen Tchetgen, 2017).

It is important to note that the validity of the total, indirect, and direct effects estimated by mediation analysis rests on strong assumptions that often, in practice, do not hold. Not only must confounders of the exposure–outcome relationship be fully measured and controlled for, but so must confounders of the mediator–outcome and exposure–mediator relationships. Because these assumptions are hard to verify, best practice requires that sensitivity analyses are performed to determine how strong an unmeasured confounder would have to be to reduce estimates of direct and indirect effects to null (or some other change point of interest; Imai, Keele, and Tingley, 2010; Imai, Keele, and Yamamoto, 2010; VanderWeele, 2015). A final assumption is that there be no mediator–outcome confounders that are affected by the exposure.

## 4 Applications to date in the literature

These approaches are beginning to gain traction in epidemiology more broadly (Visontay et al., 2022, Watkins, 2019), but the application of these methods to the field of developmental cognitive neuroscience is unclear. To assess the extent and nature of their application to date, a search of the PsycINFO and Medline databases was performed in January, 2024, combining terms related to 1) children and adolescents; 2) neuroimaging data; 3) longitudinal research; and 4) the causal inference methods of interest (see Supplementary materials for search terms). 335 papers had their titles and abstracts screened, with full-text screening for 17 of these. Results were supplemented with one additional paper that did not appear in the search because the causal inference methods were only presented in the supplementary materials.

Just eight longitudinal, neuroimaging studies involving children or adolescents were found that applied the specific approaches outlined above for improving causal inference. Methods based on propensity scores were the most popular, but there was also one paper employing targeted maximum likelihood estimation and another employing causal mediation (see Table 2). See Supplementary materials for expanded descriptions of each included study.

**Table 2:** Studies identified in database searches.

**Citation**	**Causal inference method**	**Imaging type**	Exposure and outcome* (age at outcome measurement)
(Baker et al., 2020)	IPWs + Causal mediation	Resting-state fMRI	The extent to which brain connectivity between the frontoparietal network and right precentral/frontal gyrus mediates the effect of prenatal acetaminophen on ADHD in children (age 9–11 yrs)
(Foix-L’Hélias et al., 2008)	PS adjustment	Cranial ultrasound	The effect of antenatal corticosteroids on white matter injury in preterm infants (neonates)
(Kojima et al., 2023)	PS matching	sMRI (T2-weighted)	The effect of surgery in preterm infants on total brain volume, surface area, sulcal depth, gyrification index, curvature, and a composite MRI abnormality score (at term)
(Lees et al., 2020)	PS matching	sMRI (T1-weighted) and resting-state fMRI	The effect of prenatal alcohol consumption on cortical and sub-cortical volume, cortical surface area, cortical thickness, and resting-state functional connectivity in children (age 9–10 yrs)
(Rozé et al., 2021)	PS matching + IPWs	sMRI (T2-weighted) and dMRI	The effect of maternal amino acid intake on white matter surface area, gray matter surface area, and fractional anisotropy in preterm infants (at term)
(Yang et al., 2023)	PS matching	sMRI and resting-state fMRI	The effect of social jetlag on gray matter volume and functional connectivity in children (age 11–12 yrs)
(Zhang et al., 2022)	PS matching	dMRI	The relationship** between persistent vs ‘optimal outcome’ autism in white matter abnormalities (fractional anisotropy, radial diffusivity, axial diffusivity, and mean diffusivity) in children (∼12.5 yrs at follow-up)
(Zou et al., 2021)	TMLE	sMRI (T1-weighted); Ultrasound of head circumference, sMRI, sMRI (T1-weighted)	The effect of maternal folate levels on total brain volume and cerebral white matter volume in children (age ∼10 yrs); The effect of maternal folate levels on total brain volume trajectory (across third trimester, age ∼7 yrs, and age ∼10 yrs)

PS=propensity score; IPW=inverse probability weight; TMLE=targeted maximum likelihood estimation; sMRI=structural MRI; fMRI=functional MRI; dMRI=diffusion MRI

*Note that these were often not the only or primary aims of studies, but the ones for which both causal methods and neuroimaging were employed

**Direction of causality under investigation was unclear

Five of the full-text screened studies purported to use causal mediation analysis but did not carry out the additional analyses and explicit assumption declarations that distinguish regular mediation from causal mediation. As such, these studies were not included in the current review. Even among the included studies, three applied ‘causal inference methods’ while failing to fulfill more fundamental criteria for establishing causal relationships – namely the correct temporal order of variables (Hill, 1965). Specifically, predictors must be measured before the outcome, and similarly, for causal mediation, exposure must be measured before the mediator, which in turn must be measured before the outcome.

This review identified very few relevant studies, indicating that causal inference methods have gained very little traction in the field of developmental cognitive neuroscience. Additionally, no studies were found that appropriately applied these methods to research questions where neuroimaging variables served as the predictor. Several causal inference methods introduced earlier were not found at all, for example marginal structural models and other G-methods that are used in complex longitudinal designs. Interestingly, there was also only one application of causal mediation analysis identified, despite its clear potential for application in this field (for example, structural or functional brain features serving as mediators between an exposure and outcome or serving as long-term outcomes when related to a distal exposure and more proximal mediator).

## 5 Future opportunities and directions

There is clearly great unmet potential in the field of developmental cognitive neuroscience for the application of statistical methods to enhance causal inference. Particularly lacking from research thus far are the use of these methods applied to complex, multi-wave data with repeated neuroimaging assessments across development. While this may reflect a limitation of the data that have been available to date, the recent advent of large, longitudinal cohorts with multiple neuroimaging assessments such as ABCD, NCANDA, and IMAGEN represent promising opportunities.

Studies conducted within the field of developmental cognitive neuroscience may not always be interested in inferring causality. The use of these methods for studies focused on classification or prediction, for example, is unnecessary, as these studies are not focused on the generation of causal knowledge (Danks and Davis, 2023). Causal inference is, however, instrumental to studies focused on etiological modelling, as well as studies focused on identifying targets for prevention and intervention. Greater use of statistical methods that enhance casual inference may therefore move the field of developmental cognitive neuroscience closer towards an etiological understanding of brain and behavior, as well as novel prevention and intervention strategies.

It should be reiterated that the statistical methods introduced in this review are not without their limitations – most notably, they are valid only if several strong and often unverifiable assumptions are met. Being mindful of these assumptions when designing large cohort studies is crucial going forward. For example, investigators on the HEALthy Brain and Child Development study – aware that substance-using pregnant mothers likely have very different covariate profiles to those not using substances, thus threatening the positivity assumption – are actively monitoring covariate overlap among individuals as enrolment progresses (Si et al., 2024). Also at study outset, the use of auxiliary techniques to assist with the appropriate choice and measurement of *all* relevant confounders is gaining traction, such as the construction of DAGs to visually depict assumed relationships between variables. For application after main analyses, sensitivity tests to quantify robustness to possible unmeasured confounding, similar to those described for causal mediation analysis, are also gaining popularity (e.g., ‘E-values analysis’; VanderWeele and Ding, 2017), and can be combined with others such as negative control testing (Lipsitch et al., 2010). Crucially though, without thoughtful application based on subject matter expertise and thorough methodological understanding, causal inference approaches are prone to improper use – which is clear even among the papers included in this review.

In contrast to the statistical methods reviewed above, design-based approaches (natural experiments or quasi-experimental approaches) have the potential to control for unknown and unmeasured confounders, and include regression discontinuity, difference-in-difference, instrumental variables, and within-family designs. These approaches have been comprehensively discussed previously (Ohlsson and Kendler, 2019), including their application to neuroscience and behavioral research (Marinescu et al., 2018), and were therefore not the subject of the current review. Together, these design and statistical approaches to enhance causal inference significantly broaden possibilities for addressing causal questions in developmental cognitive neuroscience.

Ultimately, these different methods for enhancing causal inference (including randomized controlled trials, as well as statistical methods and natural experiments) have unique strengths and weaknesses. This means that single studies employing these methods in isolation will never be sufficient. As a result, the route forward lies in “triangulation” of evidence (Munafò and Davey Smith, 2018). That is, assessing the convergence of results derived from several different causal approaches with complementary underlying assumptions, advantages, and limitations (see Visontay et al., 2022 for an even broader overview of causal inference methods). Similarly, evidence accumulation across diverse settings and populations is crucial to developing confidence in generalizability/external validity of findings, e.g., via cross-cohort comparison.

The current review introduced statistical methods that allow researchers to extract more valuable information from observational data as a means of promoting causal inference. To date, there has been limited translation of neuroscientific findings into effective prevention and intervention strategies, perhaps due to a lack of appropriate modelling capable of identifying relevant and replicable targets. Through the unique coupling of neuroimaging and causal modelling, the field of developmental cognitive neuroscience can move towards novel preventions and interventions that may have a greater impact on the brain and behavior.

## CRediT authorship contribution statement

**Matthew Sunderland:** Writing – review & editing, Writing – original draft. **Lindsay M Squeglia:** Writing – review & editing, Writing – original draft, Funding acquisition, Conceptualization. **Rachel Visontay:** Writing – review & editing, Writing – original draft, Visualization, Data curation, Conceptualization. **Louise Mewton:** Writing – review & editing, Writing – original draft, Funding acquisition, Conceptualization. **Hollie Byrne:** Writing – review & editing, Writing – original draft. **Emma K Devine:** Writing – review & editing, Writing – original draft.

## Declaration of Competing Interest

The authors declare that they have no known competing financial interests or personal relationships that could have appeared to influence the work reported in this paper.

## Footnotes

Appendix A Supplementary data associated with this article can be found in the online version at doi:10.1016/j.dcn.2024.101465.
