# Supplementary material for: Enhancing causal inference in population-based neuroimaging data in children and adolescents
Source: Dev Cogn Neurosci. 2024 Oct 19;70:101465. doi: 10.1016/j.dcn.2024.101465 (PMC11541429; doi:10.1016/j.dcn.2024.101465)
Supplement: Supplementary file 1 — Supplementary material [file mmc1.docx]

**Supplementary materials**

**Ovid MEDLINE search terms**

| **1**  exp case-control studies/ (1474516)  **2**  exp Cohort Studies/ (2562654)  **3**  (cohort or case control or longitudinal or prospective or retrospective or follow-up or baseline).mp. (4588151)  **4**  1 or 2 or 3 (4588151)  **5**  (neuroimag* or brain or brain imag* or magnetic resonance or functional magnetic resonance or diffusion tensor imag* or MRI or fMRI or DTI or MRS or neuropsycholog* or neurophysiolog* or electrophysiolog* or EEG or ERP or structur* connect* or function* connect* or (brain adj structur*) or (brain adj function*)).mp. (2693062)  **6**  exp Neuroimaging/ or Magnetic Resonance Imaging/ or exp Diffusion Magnetic Resonance Imaging/ (628245)  **7**  5 or 6 (2726044)  **8**  4 and 7 (466143)  **9**  exp Child/ or Young Adult/ or Adolescent/ (3862317)  **10**  (Child* or adolescen* or teen* or youth* or pediatr* or paediatr* or young or emerging adult* or youth).mp. (4842980)  **11**  9 or 10 (4842980)  **12**  8 and 11 (141410)  **13**  (causal adj2 mediat*).mp. (1365)  **14**  Propensity Score/ (15943)  **15**  (inverse probability or probability weight* or IPW or IPTW or IPCW or censoring weight* or AIPW or propensity scor* or targeted maximum likelihood estimat* or TMLE or doubly robust or marginal structural model* or marginal method* or pseudopopulation or pseudo population or generali#ed method* or G formula* or G estimat* or G computation or G method* or general formula* or structural nested model*).mp. (63283)  **16**  13 or 14 or 15 (64556)  **17**  12 and 16 (329) |
| --- |

**Detail on individual included studies**

Three studies used propensity scores to control for confounding in examining the effects of prenatal exposures on very early brain outcomes in preterm infants. Kojima et al. (2023) investigated the impact of preterm surgery on total brain volume, surface area, sulcal depth, gyrification index, curvature, and a composite MRI abnormality score, all derived via MRI in infants at term. To determine the best way to balance confounders between infants exposed and not exposed to surgery, the authors compared the performance of three kinds of propensity score-based approaches (two variations on matching as well as inverse probability weights), finding that the greatest balance was achieved with 1:1 matching (i.e., each exposed infant was matched with exactly one non-exposed infant).

Rozé et al. (2021) instead used both propensity score matching and inverse probability of treatment weighting to examine the impact of maternal amino acid intake on brain outcomes at term. Matching and weighting were performed for both a continuous and categorical maternal amino acid intake predictor, with MRI outcomes including white and gray matter surface area as well as fractional anisotropy. The authors also employed a negative control outcome analysis after matching/weighting to detect residual confounding. Negative controls (which can come in the form of a negative control outcome or exposure) substitute an outcome (or exposure) with a similar confounding structure but no plausible mechanism of effect. If the estimate is greater for the relationship of interest than for the negative control, this suggests a causal relationship. If not, confounding or other shared biases are likely responsible (Gage et al., 2016; Lipsitch et al., 2010).

Foix-L’Helias et al. (2008) investigated the effects of antenatal corticosteroids on white matter injury in neonates ascertained via cranial ultrasound, adjusting for quintiles of propensity score. These propensity scores were calculated separately within subgroups of gestational age, using generalized estimating equations to account for multiple pregnancies.

In older children, three studies used propensity score matching to control for confounding effects. Lees et al. (2020) utilised 1:1 propensity score matching as sensitivity analyses in examining the effects of maternal prenatal alcohol consumption on child brain outcomes at age 9-10. Propensity scores were calculated and used in three analyses where the predictor was considered to be either categorical prenatal alcohol exposure, a continuous drinks per week variable, or a variable reflecting temporal pattern of prenatal alcohol consumption (e.g., ‘light reducer’). Brain outcomes examined were volume, surface area, and cortical thickness in 68 cortical regions, volume in 40 subcortical segmentations, and as well as functional connectivity within and between 12 predefined networks and 19 subcortical regions.

Yang et al. (2023) examined the effects of social jetlag (the deficit resulting from a mismatch between social schedule and biological clock), corrected for oversleeping on weekends, on resting state functional connectivity within and between 12 predefined networks and 19 subcortical regions and gray matter volume in 148 regions in 11–12-year-olds. Propensity scores were generated based on a categorical exposure (<=1 hr vs >1 hr social jetlag) and used for 1:1 matching. Of note, despite the use of a ‘causal inference’ approach, both social jetlag and brain outcomes were measured at the same wave.

Zhang et al. (2022) examined the relationship between white matter microstructure abnormalities (measured at ~8.5 years) and autism outcomes (persistent autism vs ‘optimal outcome’; assessed at ~12.5 years). White matter abnormalities were explicitly referred to as the predictor and autism as the outcome, reflecting the temporal order of measurement. However, propensity scores were generated for autism outcome, i.e., the analyses performed treat autism as the predictor. Children with persistent autism, optimal outcome autism, and typically developing controls were 1:1 matched on propensity scores, with dMRI outcomes compared between groups (fractional anisotropy, radial diffusivity, axial diffusivity, and mean diffusivity).

Beyond standard propensity score-based methods, Baker et al. (2020) combined inverse probability weighting with causal mediation. The researchers examined the effects of a prenatal exposure (acetaminophen, as present in meconium) on a more distant outcome – hyperactivity at age 9-11, as mediated by resting-state functional connectivity (specifically between the frontoparietal network and right precentral/frontal gyrus). Inverse probability weights were also used to balance those exposed vs not exposed. This analysis employed some of the core features of causal mediation (e.g., making explicit the assumption of no unobserved confounders by assessing the potential effect of such confounders using a sensitivity parameter), but lacked others (the mediator and the outcome were both measured at the same wave).

Finally, Zou et al. (2021) also looked at a prenatal exposure (maternal folate levels) and its relationship with total brain volume and cerebral white matter volume in late childhood (~10 years), as well as with total brain volume trajectory across the third trimester, age ~7 years, and age ~10 years. Targeted maximum likelihood estimation was used in sensitivity analyses, where exposure was dichotomized as folate deficient vs not folate deficient.
